# Psychosocial and economic factors related to better health among older adults in Europe – a systematic review

**DOI:** 10.3389/fpubh.2026.1858548

**Published:** 2026-08-06

**Authors:** Agata Wypych-Ślusarska, Ewa Niewiadomska, Klaudia Oleksiuk, Tilman Brand

**Affiliations:** 1 Department of Epidemiology and Biostatistics, Faculty of Public Health in Bytom, Medical University of Silesia, Katowice, Poland; 2 Unit of Social Epidemiology, Department Prevention and Evaluation, Leibniz Institute for Prevention Research and Epidemiology (BIPS), Bremen, Germany

**Keywords:** European population, healthy aging, older adults, population health, psychosocial factors, social determinants of health, socioeconomic status

## Abstract

**Introduction:**

Healthy and positive aging are increasingly recognized in public health as multidimensional processes that integrate physical, psychological, and social resources to support autonomy, active engagement, and quality of life in later life. This systematic review synthesizes recent European evidence on psychosocial and socioeconomic determinants of healthy aging among older adults and contributes to the conceptualization of positive aging from a public health perspective.

**Methods:**

A systematic search of PubMed, Web of Science, and Embase identified empirical studies published in English between 2019 and 2024. Inclusion criteria encompassed studies conducted in European populations and involving adults aged 65 years and older. Studies focused exclusively on individuals under 65 years of age, samples with conditions likely to distort the examined relationships (e.g., dementia or severe mental disorders), ethnic minority groups, non-European populations, non-English publications, and other systematic reviews were excluded. Studies that included participants aged 65 years and older alongside younger age groups were retained (n = 19; 52.7%). Risk of bias was assessed using the Newcastle-Ottawa Scale for quantitative studies and the Critical Appraisal Skills Programme (CASP) checklist for qualitative designs, while two studies were evaluated using criteria consistent with the ROBINS-I framework. A narrative synthesis approach was applied.

**Results:**

Two overarching thematic clusters were identified: (1) psychosocial and economic determinants, including loneliness and social isolation, social support and social capital, socioeconomic status, autonomy and independence, and life-course context; and (2) social programs and community-based interventions. Across studies, consistent evidence highlighted the protective role of social networks, social integration, and reduced loneliness in shaping health, well-being, and cognitive functioning. Autonomy and independence emerged as central elements of positive aging, influencing perceived control, meaning in life, and overall quality of life, while engagement in social, physical, and occupational activities supported the maintenance of resources in later adulthood.

**Discussion:**

The findings underscore the need for public health policies and community environments that support positive aging through integrated physical, psychosocial, and social strategies. By emphasizing modifiable social determinants and contextual resources, this review advances the conceptual understanding of healthy aging and informs holistic, equity-oriented public health approaches for aging populations.

**Systematic review registration:**

https://osf.io/ytgqw; doi: 10.17605/OSF.IO/YTGQW.

## Introduction

One of the major achievements of medicine and public health is the increase in life expectancy (1). In the 18th century, global life expectancy was around 30 years, rising systematically only in the 20th century. By 2023, it reached 73.2 years globally and 79.1 years in Europe (2). This rise has contributed to population aging, a growing challenge for health systems and policy. By 2050, people over 65 are projected to comprise 16% of the global population (3), with Europe's older adult population expected to grow from 90.5 million in 2019 to 129.8 million by 2050 (4). While life expectancy reflects longevity, it does not account for quality of life. Healthy Life Expectancy (HLE) offers a more accurate measure by incorporating years lived in good health. In 2021, HLE was 62.2 years globally and 66.0 years in Europe (5).

Aging increases the risk of chronic diseases and disability, impacting social and economic systems. It is a priority in the Sustainable Development Agenda, which emphasizes inclusive support (6). WHO defines healthy aging as maintaining functional capacities for wellbeing (7), often linked to active or positive aging (8, 9). Key factors include cognitive and functional independence, emotional support, social engagement, physical activity, and autonomy (10). Social support improves mental and physical health, cognitive function, and quality of life (11), while personality traits influence participation. Economic factors – income, education, employment – also shape health outcomes (12, 13), though aging models often overlook subjective experiences and earlier life stages (8).

The life-course approach emphasizes continuity and accumulated influences. The Active Aging Index (AAI) includes 22 indicators across four domains: societal participation, capacity and environment, independent living, and employment (14). Active aging refers to the process of optimizing opportunities for health, social participation, and security to enhance quality of life in older age. This concept emphasizes continuous engagement in social, economic, cultural, and civic life, extending beyond the mere maintenance of physical health. Healthy aging focuses on maintaining physical and mental wellbeing throughout the life course to preserve functional ability and independence (7). It highlights the importance of preventing disease, promoting resilience, and supporting environments that enable older adults to live fulfilling lives. Although these concepts address different dimensions - participation and health – they are closely linked. Active aging cannot be achieved without a foundation of healthy aging, while healthy aging is reinforced by active engagement in meaningful social and cultural roles. Together, they represent complementary strategies aimed at maximizing autonomy and quality of life in later years. Macrosocial determinants – health programs, community initiatives, and assistive technologies – enhance wellbeing of older adults. Preventive care, chronic disease management, and digital tools like telemedicine foster independence and safety. Among the macrosocial and economic determinants of healthy aging, institutional arrangements shaping the living conditions of older adults are of key importance. These include policy frameworks addressing population aging, such as national active aging strategies and programs supporting functional independence (e.g., the development of long-term care services and assisted living). Equally significant are pension systems, including reforms affecting the adequacy of benefits and the introduction of supplementary retirement savings schemes. Another crucial factor is the availability of healthcare, particularly primary care, geriatric services, chronic disease prevention, and rehabilitation. Together, these factors constitute the structural context that can enable, or constrain, the maintenance of health and functional capacity in later life.

Current literature often isolates factors like nutrition or activity, missing the complex interplay of socioeconomic influences. There is a lack of research on diverse experiences shaped by social status, education, or housing. A holistic approach is needed to understand how psychosocial and economic factors affect aging and to guide effective strategies for improving older adults' quality of life.

This systematic review aims to examine and synthesize the evidence on the associations between psychosocial, macrosocial and economic factors and the health status of older adults (aged 65+) in European countries. In particular, the study aimed to identify how these determinants are related to functional limitations, physical health, mental health, cognitive functioning and overall wellbeing, as reported in the existing literature.

## Materials and methods

## Research question

The purpose of the work was developed according to the PICO scheme (Table 1). The PICO framework was applied in an adapted form to structure the research question. Given the exploratory and non-interventional nature of the study, the factors analyzed were conceptualized as exposures rather than interventions.

**Table 1:** Purpose of the work according to the PICO scheme.

**Population (P):**	Older adults, age 65+, all genders, living in Europe.
**Intervention (I):**	Current psychosocial factors: social support, interpersonal relationships, community participation, loneliness; macrosocial factors (accessibility to interventions and programs) and economic factors (income).
**Comparison (C):**	Studies reporting variation in exposure to psychosocial, macrosocial and economic factors.
**Outcome (O):**	Comparison of functional limitations, physical health, mental health, cognitive function, and sense of wellbeing.

Accordingly, the main research question is:

What associations are reported between psychosocial, macrosocial and economic factors in the living environment (I) and the health status of older adults aged 65 and over (P), across studies reflecting different exposure contexts (C), in terms of functional limitations, physical health, mental health, cognitive function, and sense of wellbeing (O)?

## Review protocol

The review was registered on OSF Registers. The review process was conducted by PRISMA 2020 guidelines (15) (see Supplementary File 1).

## Search strategy, sources and eligibility criteria

Search criteria of three independent researchers (AWS, EN, KO) conducted a search of three electronic bibliographic databases: PubMed, Web of Science, and Embase (between October 2024 and January 2025). The search phrase (healthy aging) AND (social determinants) AND (Europe) AND (elderly OR seniors OR old) was entered into the basic search interface of each database. Subsequently, database-specific filters were applied as follows:

• [Embase] publication years 2019 – 31.12.2024, language: English, publication type: article;
• [PubMed] publication date 2019 – 31.12.2024, full-text availability: free full text, language: English;
• [Web of Science] publication years 2019 – 31.12.2024, language: English, publication type: article.

Terms referring to older adults were interpreted as referring to individuals aged 65 years and above. The concept of “Europe” was operationalized exclusively as a keyword in the search strategy with no additional country-level restrictions applied. Additional criteria were introduced to limit the scope of the search to the period 2019–2024. This time frame was selected to capture the most recent evidence reflecting current developments in public health, including changes in social structures, health systems, and population aging, particularly in the context of recent global events such as the COVID-19 pandemic. Focusing on recent studies allowed for a more contextually relevant synthesis of psychosocial and economic determinants of health in older populations. At the same time, this decision supported the feasibility of the review while minimizing the inclusion of outdated or less applicable evidence.

Research was limited to available full-text publications written in English. No automation tools were used beyond database filtering functions.

## Selection process

**Figure 1:**
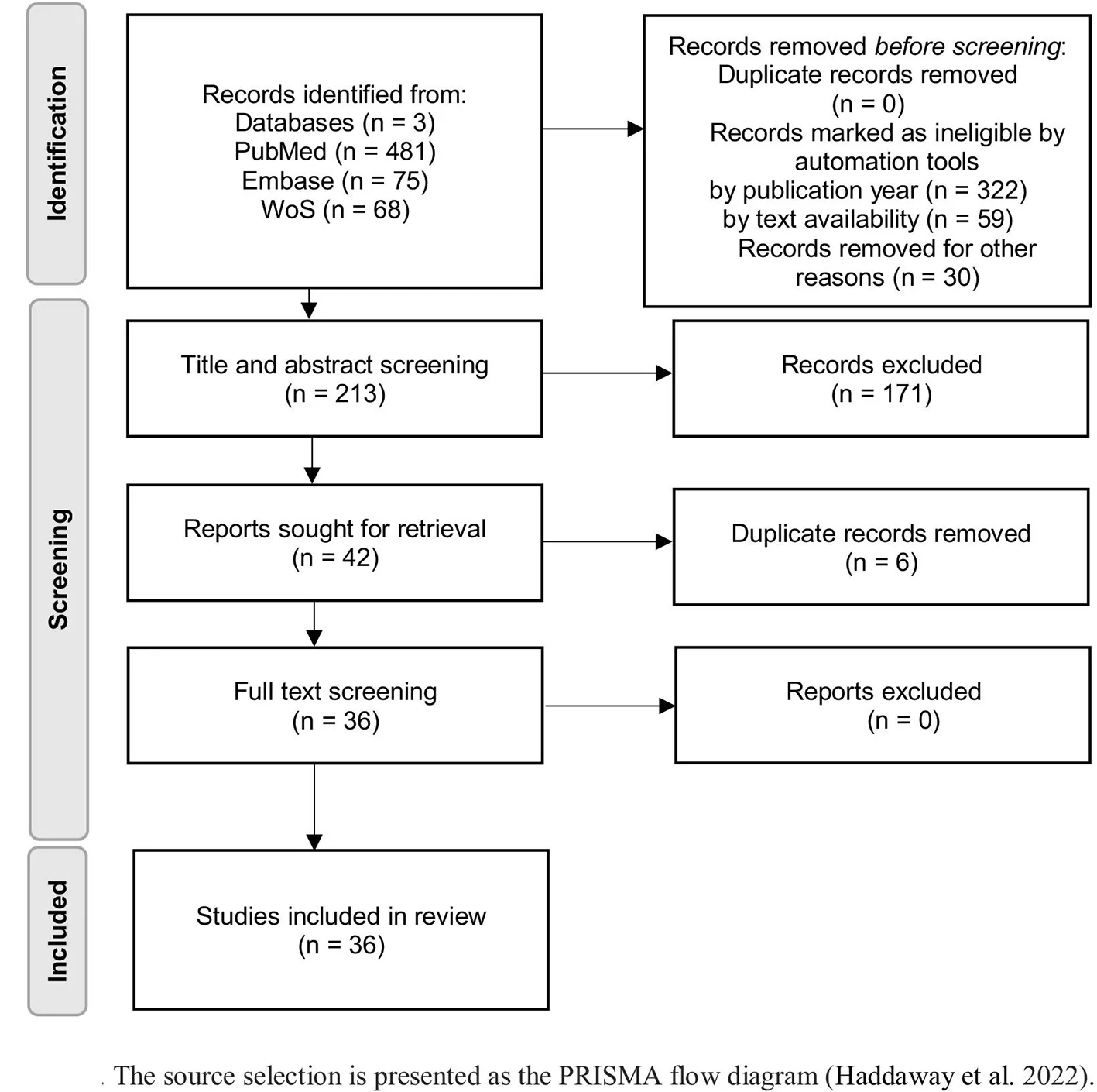
The source selection is presented as the PRISMA flow diagram (Adapted with permission from (66)).

The review's search strategy involved: (1) an automatic search (identification) and (2) a manual search (screening) – Figure 1. The automatic search was based on re-search keywords and MeSH terms, using filtering tools for electronic bibliographic databases. Three online bibliographic databases in the field of medical and health sciences: PubMed (PM), Web of Science (WoS), and Embase, were selected as the main sources. A selection of databases with an extended filtering formula was established in terms of year of issue, publication type, text availability, article language. Systematic reviews and meta-analyses were excluded. No additional hand-searching (e.g., reference list screening, citation tracking, or gray literature searching), due to the adopted assumptions, was conducted. The literature search was performed exclusively in a systematic manner, following the predefined review protocol.

After the first search stage, this study used a manual search. First, the titles and abstracts were analyzed taking into account the aim of the study (AWS, EN, KO). Then, only full-text articles were downloaded and duplicates were removed (AWS, EN, KO), and then the content was analyzed in the context of the research question (AWS, EN). A procedure was adopted whereby any uncertainties regarding selection were discussed by two authors to reach a consensus. In cases where consensus could not be reached immediately, a third author acted as an arbitrator. In practice, the review did not require the use of this procedure because there were no discrepancies that necessitated the intervention of an arbitrator. No major disagreements occurred during screening and any minor uncertainties were resolved through discussion among reviewers.

Studies were excluded that dealt with: (a) only people under the age of 65; (b) patients with conditions that could potentially affect the relationship under study (e.g., mental disorders, people with dementia); (c) only ethnic groups or national minorities; (d) only studies conducted outside Europe. Publications written in a language other than English and systematic reviews or meta-analyses were also not included in the analysis. Studies that focused on younger groups but also included people over 65 years of age were included in the analysis (*n* = 19; 52.7%). These studies most often re-ported broader age ranges (e.g., 55+ or 50–85 years), provided that individuals meeting the 65+ criterion were represented. Studies conducted in mixed geographical contexts were not excluded, provided that European populations were represented, irrespective of simultaneous data collection in non-European regions (*n* = 3; 8.3%). No heterogeneity analyses (e.g., Europe vs. non-Europe) were conducted; only a narrative synthesis was used.

## Quality assessment of included studies

Risk of bias was assessed for all included studies using appropriate methodological tools tailored to the study design. For quantitative cross-sectional and cohort studies, the Newcastle-Ottawa Scale (NOS) was applied, while for qualitative studies, the Critical Appraisal Skills Programme (CASP) was used (16, 17). Two studies were not assessed using standard tools (NOS or CASP) due to their unique methodological designs. The study by León, which employed structural equation modeling, was evaluated based on model fit quality and reporting transparency (18). For the intervention study by Naik, general criteria for assessing risk of bias in intervention studies were applied, aligned with the principles of the ROBINS-I tool (19).

Among the 36 studies assessed: 11 studies (30.5%) were rated as having low risk of bias, 14 studies (38.9%) as having moderate risk, and 11 studies (30.5%) as having high risk. The most common sources of bias included limited transparency in data col-lection in qualitative studies and potential selection bias in cross-sectional studies. The evaluation results are presented in Supplementary File 2.

## Synthesis methods

This systematic review used a narrative approach to qualitatively analyze selected scientific publications on the impact of social support on the health of older people. Narrative synthesis was conducted using a thematic approach combining both inductive and deductive elements. Initial categories were based on predefined determinants (psychosocial, macrosocial and economic factors), while themes were further refined through iterative comparison of the included studies. Themes were identified based on recurring patterns across studies and validated through discussion among the authors. Coding was conducted independently by two reviewers using a hybrid inductive-deductive approach. Initial codes were generated based on predefined categories (psychosocial, macrosocial, and economic factors) and further refined through iterative comparison across studies. Discrepancies in coding and theme identification were resolved through discussion until consensus was reached.

The analytical approach was informed by the theoretical framework of social epidemiology, which emphasizes the interconnected and multifactorial nature of health determinants. In this perspective, psychosocial, socio-economic and structural factors are understood as interacting and mutually reinforcing, rather than independent variables.

Accordingly, the synthesis was conducted in an integrative manner, without strict separation of qualitative and quantitative evidence. Both types of studies were treated as complementary, contributing to a comprehensive understanding of population-level patterns as well as lived experiences and contextual conditions influencing health and wellbeing.

This approach was particularly relevant in the context of aging research, where complex interactions between individual, social and environmental factors are central to understanding wellbeing in later life. Within this framework, a life-course perspective was also implicitly adopted, acknowledging that health outcomes in older age reflect cumulative exposures and interactions across the lifespan.

In addition, the analysis was informed by concepts derived from the ecological model, which guided the interpretation of relationships between individual, social and structural determinants of health.

## Ethical approval

No ethical approval was required, as the review was based exclusively on secondary data.

## Results

### Study selection

A total of 624 records were initially identified through database searches. Following the application of automated exclusion criteria, 322 records were removed based on publication year and 59 based on text availability. An additional 30 records were excluded for other reasons (e.g., duplicates, incomplete metadata). The search procedure (stage one) yielded a total of 213 publications: 171 (PM) + 21 (WoS) + 21 (Embase) (Figure 1). Based on screening of titles and abstracts, a preliminary selection was made to exclude publications that did not meet the inclusion conditions, resulting in 42 publications for further analysis. The next stage of qualification consisted of verification of three databases with preliminarily included literature items by one researcher (AWS). This verification allowed the rejection of six duplicate publications. This resulted in 36 publications that were considered for further inclusion. A further selection was made by reading the entire text by three independent researchers. No more articles were rejected at this stage.

## Study characteristics

The results of the study were presented, taking into account social, economic, and environmental factors. Potential mechanisms for the impact of social support on the health of the older adults were also analyzed. Table 2 presents the literature cited in the article, organized according to broad subject areas addressed across the manuscript. Its purpose is to provide a structured overview of the conceptual and empirical background underlying the study, rather than to summarize the included studies themselves. Therefore, the categorization used in Table 2 does not directly correspond to the structure of the Results section or the narrative synthesis. While the table groups references into distinct thematic areas for clarity, the narrative synthesis adopts a more integrative approach. This reflects the complex and interconnected nature of the analyzed determinants, particularly in the context of social epidemiology, where factors such as psychosocial and socioeconomic conditions overlap and interact.

**Table 2:** Literature cited in the article divided according to the subject area.

Context/section	Description	References
General information	• The life expectancy of the population; • Healthy life expectancy; • Challenges for health and social care systems; • Promoting and supporting healthy aging; • Definition of healthy aging; • Socioeconomic determinants of healthy aging	(1–4) (5) (3, 29) (7) (8, 9) (8–14, 62)
Loneliness and social isolation	• The importance of loneliness and social isolation; • Mechanisms of the relationship between loneliness, social isolation and chronic diseases, general health; • Social engagement and family relationships as determinants of health	(20, 56) (16, 26, 29) (24, 26–32)
Social support and involvement	• Social support definition; • The relationship between routineness of social and family interactions and well-being, health-related quality of life; • The impact of social support on reducing health inequalities	(8, 34) (8, 9, 31, 37–48) (43)
Socioeconomic status, adaptation, and independence	• Socioeconomic status and physical and mental health of older people; • Extending working life and the sense of independence; • The importance of the external environment in shaping autonomy in healthy aging; • Acceptance of limitations resulting from aging and emotional stability	(38, 44, 46, 49–53) (52) (54, 55) (8, 26, 31, 41)
Life-course perspective	• Past Events as the basis for current situation and life satisfaction; • The relationship of difficult childhood experiences to phenotypic aging	(8, 23) (23, 57)
Social interventions and programs	The importance of community programs for healthy aging	(50, 58–61)

A comprehensive overview of the included studies, including study design, country, sample size, population characteristics, and main findings, is provided in Supplementary File 3 (List of publications included in the systematic review).

## Narrative synthesis

A total of 36 studies were included in the review, comprising a diverse range of methodological approaches, including cross-sectional (*n* = 15), cohort (*n* = 11), qualitative (*n* = 7), and other designs such as structural equation modeling and intervention studies (*n* = 3). The studies covered a wide geographical area, encompassing both single-country analyses (e.g., Spain, Italy, Sweden, Germany, Portugal) and multicountry datasets, particularly from large-scale surveys such as SHARE, as well as selected studies including non-European populations alongside European samples. Study populations primarily consisted of older adults aged 65 years and above, although several studies included broader age ranges (e.g., 50+ or 55+) while retaining representation of the target population.

Across the included studies, a wide spectrum of psychosocial, socioeconomic, and structural determinants was examined, with particular emphasis on social relationships, social support, socioeconomic status, and environmental context. These factors were analyzed in an integrated manner, reflecting their interdependent and mutually reinforcing nature within the framework of social epidemiology.

The findings are presented below as a narrative synthesis structured around key thematic domains. This approach integrates evidence across studies to identify recurring patterns and relationships, rather than reporting individual study results separately.

## Psychosocial and economic factors

### Loneliness and social isolation

One of the psychosocial determinants of health is loneliness, identified by the WHO as a global public health threat (20). Numerous studies confirm its role, along with social isolation, in the incidence of various conditions (21–23). Though distinct, loneliness and social isolation are linked (21). Loneliness is a subjective feeling arising from insufficient social contact or unmet expectations in relationships, often due to isolation or lack of trusted ties (22). Social isolation is objective, defined by a person's social situation and lack of contacts (23). Their relationship can be reciprocal: isolation may precede loneliness, often during bereavement, especially among older adults, or result from prolonged loneliness (21). Psychological, behavioral, and biological concepts explain their impact on health (21, 22). Stress mechanisms may lead to mental disorders, while unhealthy behaviors linked to loneliness and isolation cause biochemical changes in the body.

In a cohort study, Christiansen et al. (21). showed that loneliness and social isolation independently increased the risk of type 2 diabetes by 98% and 56%, and cardio-vascular disease (CVD) by 30% and 24%, respectively. Indirect mechanisms included negative affect, insufficient sleep, and obesity for loneliness, and physical inactivity and poor sleep for isolation. Stress explained over 40% of the link between loneliness and CVD. Lonely and isolated individuals may cope poorly with stress, resorting to unhealthy behaviors like smoking. These factors influence disease risk through distinct pathways. The COVID-19 pandemic (2019–2023) enabled research on social isolation and health. In many European countries, restrictions confined older adults at home, limiting contact and activities. A British study during the first lockdown phase (March – June 2020) highlighted the role of social capital, such as neighborhood ties, when family and friend contact was severed (24). A holistic health approach must include psycho-social aspects. Healthy aging depends on the quality of social interactions. Research shows social relationships are a key psychological need and vital to wellbeing in old age (24–26). They help counteract risks of CVD, depression, cognitive decline, and sleep problems (24). Studies included in this review indicate that beyond family, neighborhood ties engage seniors in activities, foster community integration, and serve as social capital during difficult times (26).

Brown and Reid (24), conducting qualitative interviews with people over 70, found that neighborhood relationships were key to wellbeing during the COVID-19 pandemic. Isolation encouraged seniors to strengthen these ties and meet new neighbors. Social engagement boosted feelings of security, especially among those living alone and cut off from family. Neighborhood initiatives – flyers, online groups, balcony conversations – fulfilled the need for contact, reduced loneliness, and mitigated isolation. The study highlights the overlooked health value of simple social interactions. These build neighborhood ties and protect against loneliness. A Swiss study (26) confirmed the importance of local communities. Interviews with 12 older adults identified key health areas, including social belonging and support networks. Functioning in local communities was a health determinant. Social participation and relationships are social capital that seniors can rely on in times of need. Supportive neighborhoods protect against loneliness and promote healthy behavior (27). In the Netherlands, older women were motivated by social pressure from engaging groups to stay active through walking, exercise, and recreation (28).

Many studies also emphasize the importance of family ties and especially partnerships in healthy aging (29–31). Being married or in a partnership supports cognitive function, reduces the risk of dementia, and can influence social activity by engaging in networks of relationships, friendships, and contacts specific to each partner (32). People in relationships have been shown to have higher levels of social integration than those who are single. The amount and types of social ties and contacts have a stimulating effect on brain function and the maintenance of cognitive reserve. In addition, people in relationships benefit from social support in emotional, instrumental, or material terms. The source of this support is often a life partner or spouse (30). Being in a relationship further promotes healthy behavior. Partner status influences engagement in socially productive recreational activities, which can contribute to reducing the risk of dementia and support cognitive reserve. These behaviors include not only physical activity in the form of daily walks, bicycle rides, and simple exercise done at home or in the community, but also mental activities such as reading books, solving crossword puzzles, or rebus. The loss of a partner and a period of widowhood often means a loss of this social capital and can also affect cognitive decline.

An analysis of SHARE (Survey of Health, Aging, and Retirement in Europe) survey data, including 215,989 people over 50 from 20 European countries, found that the lack of a stable partnership after divorce or widowhood can negatively affect cognitive health in old age (30). In contrast, re-partnering has a protective effect against adverse cognitive trajectories. These effects are more pronounced in those with less education. Another analysis from the 6th wave of the SHARE study conducted in 2015 in Spain, involving a group of 5,583 older adults (mean age 70.28 ± 10.55 years), yields similar observations indicating that feelings of loneliness (measured using the Revised UCLA Loneliness Scale) are a major determinant of physical, emotional, cognitive, and functional health (29). Higher functional abilities were observed for those who did not experience loneliness compared to those who did (mean score 96.03 vs. 91.47, *p* < 0.001). Differences were also noticeable concerning the number of social contacts. A higher functional ability score was found for those with 1 to 7 friends (95.56) compared to those who indicated no social contacts (93.91, *p* < 0.001). Feelings of loneliness also affected cognitive and sensory abilities, which were lower among those reporting some degree of loneliness (46.97 vs. 54.19, *p* < 0.001) (29).

The importance of social contact and the impact of loneliness on wellbeing are highlighted by a Q-method study of 53 - older adults in the Netherlands (31). Respondents ranked 34 opinions and explained their choices in interviews. Next to health and security, a life partner was most valued for their constant presence and emotional support. The absence of such support may pose challenges individually and systemically. A study in Salerno among non-agenarians and centenarians showed varied living arrangements, with family or third-party support contributing to healthy aging (32). Regular visits are crucial for those living alone. A cohort study of 25,356 people explored how loneliness and social engagement relate to C-reactive protein (CRP) levels (33). A reciprocal loop was found: higher CRP correlated with loneliness and lower engagement, while engagement reduced CRP. Social activity lowers stress and inflammation, and emotional support reduces mental health risks. Physical activity linked to social engagement also helps reduce CRP. Gao et al. (33) exemplify the link between bio-logical mechanisms and social health determinants.

A synthetic analysis of the collected studies indicates consistent and recurring patterns observed across the reviewed literature. Loneliness and social isolation affect the health of older adults through complex indirect mechanisms involving psychological, behavioral, and biological factors. Older individuals are particularly vulnerable to experiencing loneliness, especially in the context of the loss of close relationships, including life partners, which may further contribute to the deepening of social isolation. Loneliness may lead to reduced social contacts and lower levels of activity, while increasing social isolation has a negative impact on both physical and mental health. Limited activity and restricted social relationships are associated with a higher risk of non-communicable diseases, as demonstrated in the studies discussed above. In this context, social support may serve as a key protective factor, mitigating the adverse effects of loneliness and social isolation and supporting healthy aging. The mechanisms through which social support operates are examined in detail in the following section.

### Social support and involvement

Through social networks and community involvement, another element of social capital is created - social support. It is also crucial in shaping the health of older adults. There is no single definition of social support; it is generally understood as an individual's perception of being needed, loved, and valued within social relationships (34). Social support is built through an individual's interactions and social involvement. In the case of seniors, social resources tend to be lower, making social interactions less intense as well.

According to the theory of social-emotional selectivity, older people will prioritize maintaining and engaging in social interactions that are emotionally meaningful (35). In line with this theoretical perspective, findings from the included studies suggest that social support plays an important role in shaping health outcomes, particularly by buffering the effects of stress and supporting emotional and physical needs. Social ties are formed by nurturing these relationships, and social support may be the next step. The latter is an important social factor influencing health, reducing the effects of stressful events. It is an instrumental value through which individuals can satisfy physical and emotional needs. Older adults often have limited social capital and increasingly scarce social resources, which may result in less support. At the same time, interpersonal interactions can be marked by routine and predictable situations (36). A 2019 study of 120 people aged 65–84 living in Switzerland examined how routine interactions affect the health of older adults. The results indicated that positive affect was influenced by a higher overall routine of social interactions, a higher routine of social interactions with the same modality, and a higher routine of social interactions with the same type of partner. Moreover, evidence from the studies analyzed in this review indicates that social interactions in later life may become more routine, yet remain positively associated with wellbeing (37). Thus, the routineness of social interactions was associated with higher wellbeing (37).

Social engagement also affects health-related quality of life (HRQoL), which refers to a subjective assessment of physical and mental health status carried out by an individual or a group during a specific period of life. A cross-sectional population-based study conducted in 2019 in the canton of Basel-Landschaft, in northwestern Switzerland, brings such observations (38). Analysis of data collected from 8,786 people over the age of 75 showed that involvement in social activities was associated with improved HRQoL. Older age, female gender, the presence of multimorbidity and polypharmacy, as well as social isolation and loneliness, negatively affected quality of life (38). Informal social support was received by 36.4% of the respondents, and from at least one organization, such support is received by 30.6% of older adults. In most cases (80.7%), this support was at a low or moderate level. Lower involvement in social activities was associated with greater health problems and lower health-conditioned quality of life. Similar results were obtained by Guastafierro et al. (39) in a study of 431 older adult residents of the Lombardy region. They showed that higher scores on the Social Network Index were associated with better quality of life, while high scores on the loneliness and disability scales indicated a decrease in quality of life. Those with a good social support network had a higher level of functioning and health-conditioned quality of life. Participants living in smaller villages had a more positive view of the role of older adults in the community. They felt they belonged in the community and were treated well, respected, and supported. Participants who felt their role in society was positive had a stronger sense of belonging to their neighborhood, community, and/or family. In contrast, the results of a study of 128 older adults in Spain showed that the availability of social resources positively influenced perceptions of quality of life and improved memory (40). Participants in a focus group study of older adults in Spain emphasized the importance of community initiatives for the senior citizens (41). They pointed out that society is increasingly concerned about the older people and is organizing more activities aimed at them, including promoting intergenerational activities to better under-stand younger generations. In a 2019 survey of older adults from Estonia, Lithuania, and Latvia, associations between social support and other areas of life became apparent (42). Social support was significantly associated with higher self-rated reading (Cramer's V coefficient (VC) for Estonia/Lithuania/Latvia respectively, VC = 0.347/0.342/0.251) and writing (VC = 0.267/0.326/0.269), higher levels of memory (VC = 0.245/0.169/0.202), life satisfaction (VC = 0.133/0.162/0.134), lower levels of depression (VC = 0.146/0.166/0.140) and less loneliness (VC = 0.103/0.08/0.131), more hope for the future (VC = 0.205/0.173/0.069), less difficulty in daily tasks (VC = 0.130/0.073/0.006), fewer illnesses (VC = 0.133/0.054/0.144), and less need for help with daily tasks (VC = 0.284/0.231/0.306) [38]. These findings underscore the importance of building and maintaining strong social networks to improve the quality of life in the aging population.

Analysis of data from a cohort study of 585 people aged 60–84 in Portugal highlights the importance of social support in other areas of older people's lives, as well as its impact on reducing health inequalities to HRQoL (43). Education had a positive effect on HRQoL (β = 0.28; 95% CI: 0.05–0.48), but it was social support that explained 54% of the association between education and HRQoL (β = 0.15; 95% CI: 0.05–0.26). A similar pattern was observed for occupation. Perceived income adequacy had a total effect on quality of life (β = 2.74; 95% CI: 1.68–3.93). Thus, social support can help older adults improve their quality of life by mitigating the impact of social inequality (43). The availability of social resources in the form of supportive communities for the older adults, participation in social life, has a positive impact on self-rated health, as well as cognitive and emotional health. A study of 128 senior volunteers in Madrid points to such a relationship (40). The availability of social support positively affected quality of life, improved explicit memory, and reduced the risk of depression in older adults. The protective effect of social resources may be the result of a socially active lifestyle, which builds a sense of security from having a social network and thus protects against isolation and loneliness. The family plays a special role in building and maintaining social networks. The importance of the family for the health of the older adults must be considered in a broader context. First, the presence of others builds a sense of security, protects against loneliness, and also stimulates cognitive abilities. The latter are supported by good family relationships. Confirmation of the above statements is provided by the results of a study conducted in Germany among people over 80 years old (44). Participation in social activities, such as social gatherings and participation in clubs or organizations, contributed to better mental and physical health and also had a stimulating effect on cognitive function.

A study conducted in two of the most deprived areas of northeastern Hungary (Hajdú-Bihar and Szabolcs-Szatmár-Bereg) observed that strong social support and support in personal matters positively influenced the psychological wellbeing of the older adults (45). A perceived high level of social support increased wellbeing in people over 65 (β = 8.50, *p* = 0.002), while a higher mental stress score was associated with a lack of support in personal matters (β = 3.64, *p* = 0.008). Older adults with strong social support (β =0.84, *p* = 0.007) reported higher life satisfaction compared to those with low social support (45). In a study of 17 men aged 85–90 who were participants in the Uppsala Longitudinal Study of Adult Men, information was collected on individual under-standing of the concept of “healthy aging.” Among the social factors that determine healthy aging, men emphasized the importance of relationships with family, friends, and neighbors. Proper social interactions are key to a sense of belonging and emotional support (8). Similar observations come from a study of 53 people aged 65–93 in the Netherlands (31) and one hundred and eighty community residents aged 65–94 in Sardinia, Italy (9). Residents of the Netherlands, through structured interviews, indicated the areas that are important to their wellbeing. They placed high importance on family and proper relationships, affecting wellbeing. In the absence of a life partner, other social relationships, mainly neighborhoods, were given significant importance. They, too, provided a sense of security and the knowledge that one could count on someone's help in difficult times (31). Italians, on the other hand, stressed the importance of emotional support and positive relationships with family members on their cognitive performance and flourishing. In a psychological context, flourishing refers to a state in which a person experiences a high level of wellbeing, satisfaction with life, and full realization of one's potential, meaning that one feels fulfilled, has a sense of meaning and value in one's life, and maintains strong social ties. Hierarchical regression analysis showed that 30% of the differences in levels of flourishing could be explained by satisfaction with family relationships, psychological resilience, and metacognitive efficacy. In addition, men achieved higher levels of flourishing and were more satisfied with family ties than women (9).

A longitudinal study from Sweden indicates that low social contacts (OR = 1.88; 95% CI: 1.47–2.39), low levels of social support (OR = 1.67; 95% CI: 1.32–2.12), and low levels of social participation (OR = 1.56; 95% CI: 1.21–2.02) were risk factors for poorer health trajectories, assessed using the Health Assessment Tool (HAT) and including physical, cognitive, multimorbidity, and disability dimensions (46). Also stimulating cognitive development is the change in social role and the transition to the role of grandparents. This is indicated by the results of an analysis of data from a long-term study from the SHARE and involving a sample of 19,953 people aged 50–85 (47). Transition to the role of grandparents was associated with higher levels of cognitive functioning compared to those who did not become grandparents (β = 0.79, *p* < 0.01). Women who became grandparents made greater gains in their cognitive functioning trajectories than men or women who did not become grandparents. Becoming a grandparent can generate psychological benefits through social interactions or positive emotions, can affect feelings of being needed, stimulate social activity, and contribute to making new friends related to caring for a grandchild. Also, researchers participating in focus group study in Spain pointed to good relationships with grandchildren as a source of wellbeing (41). These relationships were mutually beneficial, according to the respondents. Participants said they had systematic contact with their grandchildren through their role as caregivers. Grandchildren were a source of joy and provided a sense of being loved and useful. Due to the declining social resources of older people related to aspects such as retirement, death of spouse, friends, and deteriorating health, institutional support also seems important. Its form can be preventive home visits, which are used by older individuals in Sweden (48). During such visits, a diagnosis can be made of the living conditions, wellbeing, and needs of older adults. A study conducted in two municipalities in north-eastern Skåne, Sweden, among 619 individuals (mean age 80.6 ± 2.2 years) identified key factors perceived by respondents as determinants of wellbeing. Respondents most often cited the ability to do things that make a person feel valuable, the absence of physical problems that affect participation in social activities, and the absence of reduced energy and impaired stamina. Older people who rated their health well were more likely to show better indicators of mental and physical functioning than those who rated their health less well. Among the mental factors were such determinants as lack of loneliness and satisfaction with life. Social contacts and physical activity were also important, ensuring proper functioning (48).

A synthesis of the collected studies indicates consistent and recurring patterns regarding the role of social support in the process of healthy aging. Regardless of the context of the studies analyzed, social support consistently emerges as a key protective factor that may mitigate the adverse effects of loneliness and social isolation. Sources of support vary, ranging from family and close relationships to the broader social environment. Importantly, older adults do not require extensive social networks to experience its benefits; even maintaining a limited number of social relationships fosters the development of lasting social bonds and a sense of belonging. As previously noted in relation to loneliness as a determinant of health, social support operates through complex indirect mechanisms. Key elements include a sense of security, awareness of the availability of assistance, and the belief that others can be relied upon in difficult situations, all of which strengthen mental health and resilience to stress. At the same time, social interactions activate individuals at both physical and cognitive levels, requiring them to engage in even minimal effort, such as leaving the house, interacting with others, or participating in everyday activities. Family relationships and transitions in social roles, such as becoming a grandparent, also play a particularly important role. These transitions involve adaptation to new responsibilities and expectations and contribute to a stronger sense of purpose, usefulness, and security. From this perspective, the organism and individual functioning are not static but remain active through social engagement and participation, which constitutes an essential component supporting healthy aging.

### Socioeconomic status, adaptation and independence

Socioeconomic status (SES), adaptation, and independence are closely linked, affecting various aspects of an individual's life. Higher SES is often associated with better access to resources such as education, health care, and professional opportunities, which can lead to greater independence. The same can be observed for adaptation, or an individual's ability to adjust to changing life circumstances. Evidence from the included studies suggests that individuals with higher SES often have better resources, such as access to education, social support, and financial stability, which in turn can support their ability to adapt. The latter is one component of better coping with stress, dealing with life challenges, and building an individual's independence. Results from the longitudinal Swedish Study of Aging and Caregiving in Kungsholmen (SNAC-K) suggest that the health of older adults is partly shaped by material factors, such as income and assets, which allow people to secure the goods and services needed for a healthy life (e.g., housing, food, health care), but also by the intangible resources and abilities provided by education (46).

Financial hardship (OR = 4.33, 95% CI: 1.45–12.99) was a factor that increased the risk of poor health. Studies also indicate that higher SES is associated with the maintenance of cognitive function in old age. The results of a German study in a population over 80 years old showed that higher education was associated with less cognitive decline in the DemTect subtests of learning a list of words, transcoding numbers, and repeating numbers in reverse order (44). A study of 6,735 older people analyzing the relation-ship between socioeconomic status and C-reactive protein (CRP) in England yields interesting results on the relationship between SES and health status (49). The study found that respondents with a high school education (β = −0.033, *p* < 0.05) or higher (β = −0.127, *p* < 0.05) had lower levels of CRP. In addition, higher wealth was correlated with lower CRP levels (moderate wealth: β = −0.069, *p* < 0.05; high wealth: β = −0.125, *p* < 0.05).

Financial stability provides a sense of security and choice, which contributes to maintaining independence and health. People with higher incomes have better access to healthy food, health care, and recreational activities. A 2019 study of 8,786 people over the age of 75 in northwestern Switzerland found a link between SES and quality of life (38). Better socioeconomic status, higher income, possession of additional insurance, and higher levels of education were associated with better quality of life among older people living at home (38). Some analyses considering aspects of SES and health go a step further by focusing on potential health inequalities between men and women (50). The roles and professional positions held throughout life may determine the amount of financial resources available in later life, with studies indicating that opportunities for financial independence are lower for women. Women live longer than men, but already healthy life expectancy rates do not show such significant differences. Women's continued life, especially in old age, can be fraught with economic deprivation. An analysis by Chen et al. (50) revealed specific gender differences in key domains of social aging in different OECD countries. In each country, gender differences in key aspects of social aging favored men. Gender differences were particularly pronounced in the domains of productivity and engagement, safety and cohesion, where men had an advantage (mean difference 10.2, 95% CI 7.8–12.6; *p* < 0.0001 for productivity and engagement, 10.3, 7.8–12.7; *p* < 0.0001 for safety, and 21.1, 13.9–28.1; *p* < 0.0001 for cohesion). Although the domains of wellbeing and equality showed more mixed results, they still showed a slight advantage for men. The study underscores the need to even out economic differences between the sexes across the lifespan, as such strategies can have a measurable impact and influence better health indicators for women in older age. Successful, healthy aging is therefore a social domain. Economic resources are influenced by work and the ability to continue working after retirement. Data from the Helsinki Birth Cohort Study also highlight similar differences (51). At age 65, the probability of being healthy was 47.8% in men and 41.9% in women; at age 75, 27.5% and 27.1%, respectively. Higher socioeconomic status in childhood, longer time in education, and being ever married were associated with a higher probability of healthy survival in women (51).

A study conducted on the incentives and experiences of extending working life be-yond the expected retirement age of 65 among Swedes indicated that work contributed to the maintenance of internal resources such as cognitive function, physical fitness, and greater vitality, as well as to the maintenance of external resources, which also include economic benefits, a sense of being needed, and an increased sense of purpose in life (52). Thus, the ability to remain professionally active translates not only into tangible financial benefits but also, through the operation of the factors already described (social support, social interaction), into building a sense of independence, vitality, and a better assessment of one's health (52). The financial aspect was an important factor for engaging in labor force participation, especially for the study participants who did not have a high pension. The additional source of income meant that they could maintain a certain standard of living, travel, get better groceries, or save money, which simultaneously boosted their wellbeing. Work also helped maintain structure in their lives, bringing routine and organization, which in turn helped keep them active and vital. Being active, taking on new challenges, meeting people, and having to learn also had an activating effect on cognitive function, increased independence, self-confidence, and overall satisfaction. Participants in the study, moreover, felt that they were still needed, and this in turn affected their sense of meaning in life. The results of this study indicate that work is not only related to the economic dimension, which is important for being able to fulfill various needs, but in the case of older adults, it is primarily important in building social resources and maintaining physical, cognitive, and mental activity and vitality. The results of the international cohort study, based on data from five nationally representative surveys in 24 countries: Health and Retirement Study (HRS), English Longitudinal Study of Aging (ELSA), Survey of Health, Aging and Retirement in Eu-rope (SHARE), China Health and Retirement Longitudinal Study (CHARLS), and Mexican Health and Aging Study (MHAS) unequivocally emphasize the importance of SES as a risk factor for depression and the role of social activity and loneliness in this relationship (53). The study included individuals over 50 years of age, without diagnosed depression and symptoms of psychiatric disorders. During the five-year follow-up, of the 69,160 participants, 20,237 developed depression. Those with low SES had a higher risk of depression (HR = 1.34; 95% CI 1.23–1.44) compared to those with high SES. An additional significant factor mediating the association between SES and depression was loneliness and social inactivity. In the context of the analysis by Bjuhr et al. (52), it is important to highlight the importance of the results, suggesting the introduction of integrated approaches that consider SES, social activity, and loneliness to reduce the risk of depression in the older population.

Multidimensionality in relation to SES, social integration, and health is evident in the results of the prospective population-based Heinz Nixdorf Recall Study (HNR), conducted in three major cities in the Ruhr region (Bochum, Essen, Mülheim) in West-ern Germany (54). It focused on the relationship between neighborhood characteristics and the health status of residents. It was hypothesized that certain neighborhood characteristics could influence behavioral responses, perceptions, and health behaviors, thus shaping the population's health profile. Attributes such as leisure activities, public transportation connections, economic prosperity, social trust, social networks, and public safety affect the quality and SES of a neighborhood. Neighborhoods with low SES may exhibit less favorable characteristics compared to neighborhoods with high SES. In the context of older adults, the neighborhood of residence can be an important determinant of health. Seniors are less likely to be mobile and generally organize their activities at or near their place of residence. Thus, unfavorable neighborhood conditions low SES of the neighborhood can negatively affect the health of residents. The study found that low neighborhood SES was associated with lower physical activity among older residents, due to less pronounced social networks, trust, and social sup-port. Neighborhood SES also affected an individual's adaptability to adverse conditions, exposure to environmental stressors, and availability of physical and social re-sources. The mental health of older adults depended on certain neighborhood characteristics, such as noise, litter, neglected houses and disorder. A survey of 112 healthy people aged 60+ from the south of Ireland also highlights the importance of the neighbor-hood of residence in promoting physical activity, specifically walking (55). Respondents indicated their preference for walking. Most preferred outdoor spaces that offered variety and greenery. Also important was the diversity of the neighborhood, which could have a stimulating effect on cognitive function. In contrast, those who avoided such stimulation preferred walking in quiet neighborhoods. The analysis showed that those who reported a higher frequency of cognitive failures were less likely to walk in areas characterized by the presence of people (rho = −0.25, *p* = 0.02).

Socioeconomic situation, whether in the individual dimension or in relation to the characteristics of the area of residence, is one of the elements that affect the independence of older people. A study conducted in the Netherlands in a group of people over 65 years of age provided insight into the importance of particular aspects of life in shaping good health (31). Among other things, participants in in-depth interviews pointed to independence as an important determinant of wellbeing. Many people did not want to be forced to ask for help from others and to control the situation themselves. This was sometimes argued by the fear that a request for help would be rejected. At the same time, respondents stressed that maintaining mental health was one of the conditions for being independent and making autonomous decisions (31). A sense of control over one's health was also found to be a significant factor in psychological wellbeing and higher life satisfaction in the Helsinki cohort (45). The results of a population-based study from the Uppsala Longitudinal Study of Adult Men from Sweden also highlight the importance of independence and autonomy in healthy aging (8).

In structured interviews conducted, the men indicated areas important to their wellbeing. Adaptation, in their view, meant simultaneously accepting the processes and changes resulting from aging and coming to terms with physical and cognitive limitations. At the same time, it was the same as adopting new strategies for coping and daily functioning. Adaptation can thus be a stimulus to action to prevail and feel independent in new life circumstances. Also, participants in a focus group study conducted in Girona, Spain, acknowledged that the ability to accept limitations was an important aspect of their wellbeing (41). Adjusting to mobility limitations and to physical and social changes leads to emotional stability. Similar observations came from a study con-ducted in Switzerland (26). Content analysis based on data from semi-structured inter-views identified areas of health needs for older individuals, among which independence and autonomy were key. These meant the ability to live and manage independently. At the same time, respondents indicated that accepting help from others was a sign of dependence and was sometimes treated as shameful. Concerns about a lack of independence overlapped with health concerns, especially the risk of dementia.

The findings demonstrate clear interconnections between socioeconomic status, adaptation, and independence, and the previously discussed determinants of health, such as loneliness and social support. These factors form an interrelated system of dependencies in which economic resources act as a foundation enabling individuals' activation across multiple domains of functioning. Higher socioeconomic status provides greater access to both material and non-material resources, translating into better living conditions, higher quality of the residential environment, and increased opportunities to access healthcare and engage in social activities. Consequently, it supports not only the maintenance of health but also the development of individual independence and autonomy. Financial resources expand opportunities for participation in social life, thereby indirectly reducing loneliness and social isolation, previously identified as significant health risk factors.

At the same time, the findings suggest that socioeconomic status operates through indirect mechanisms, including the ability to adapt to changing life circumstances, cope with stress, and maintain social engagement. A higher level of resources promotes an active lifestyle that encompasses both physical and cognitive activity, which is essential for maintaining functional capacity in older age. It is important to emphasize that not only material factors but also their psychosocial dimension are relevant. Economic stability strengthens the sense of control over one's life, security, and agency, which directly contributes to psychological wellbeing. In this context, independence and autonomy are not merely outcomes of available resources but represent key components of healthy aging, enabling individuals to actively participate in social life and make decisions about their own lives. Conversely, lower socioeconomic status may limit opportunities for social participation, increase the risk of loneliness, and contribute to poorer health outcomes, reflecting the previously observed relationships between social isolation and health. This clearly demonstrates that economic, social, and psychosocial determinants do not operate independently but rather interact and reinforce one another.

From a broader perspective, socioeconomic status, adaptive capacity, and the level of independence constitute key elements of the healthy aging process, shaping both living conditions and levels of activity and social integration. Their influence is dynamic and multidimensional, fitting within the broader framework of social determinants of health.

### Life-course perspective

The life-course perspective is considered here as an integrative framework linking the cumulative effects of different determinants across the lifespan.

One of the criticisms leveled against theories of healthy aging is that they do not consider a life-course perspective, only analyzing the determinants of population aging at a specific point in life (8). Meanwhile, overall wellbeing cannot be fully understood without considering earlier life stages and life events. Interviews with a group of older men from Sweden highlighted the importance of past events on perceptions of the current life situation (8). The respondents referred to experiences from their child-hood, adolescence, starting a family, and working life and indicated that these events provide an important framework for their current situation and life satisfaction. They particularly emphasized the importance of happy, good relationships, meaningful work, and good finances. These memories mitigated the potential hardships of old age associated with illness, reduced fitness, and declining vitality. Studies also indicate that healthy aging is a combination of genetic, environmental, and behavioral factors (23). In addition to the classic determinants of health, a life-cycle perspective appears to be important, especially considering childhood experiences. This is a critical developmental phase that can significantly affect physical health, mental health, and psychosocial functioning in adulthood. Experiences of emotional neglect or abuse in childhood can lead to permanent changes in brain structure, the functioning of physiological systems, and metabolic processes. These changes can have long-term negative effects on health, shaping adverse health trajectories over a lifetime. Zhang et al. (56), using data from the UK Biobank covering nearly 160,000 individuals, conducted comprehensive analyzes to examine the links between levels of emotional support in childhood and dis-eases and characteristics of aging. The study found a strong correlation between levels of CES (Cognitive Emotional Support) and the rate of aging. Higher levels of support contributed to slower acceleration of biological age and also influenced lower levels of CRP. In addition, those receiving emotional support in childhood had a lower risk of age-related diseases and were observed to have a slower decline in physiological function (23).

An analysis of data from the UK Biobank study (2006–2010) and the Online Mental Health Survey (2016) assessed the associations between difficult childhood experiences (physical and emotional neglect, sexual abuse, physical and emotional violence) and measures of phenotypic aging (57). The results indicated that each type of difficult childhood experience was associated with accelerated phenotypic age. Participants who experienced four (β = 0.296, 95% CI, 0.130–0.462) or five (β = 0.833; 95% CI, 0.537–1.129) difficult childhood experiences had higher acceleration of phenotypic age. The mediating elements were anti-health behaviors. The study authors suggest that individuals who experienced difficult childhood situations may be more likely to adopt unhealthy lifestyles based on learned social patterns (e.g., poor diet, smoking, or drinking).

The life-course perspective provides a key interpretative framework for understanding the processes of healthy aging, highlighting the cumulative and long-term effects of various health determinants. The findings confirm that experiences from earlier stages of life, particularly childhood, can have a lasting impact on health trajectories in older age, operating through biological, psychosocial, and behavioral mechanisms. A critical aspect in this context is the continuity of influences, adverse socioeconomic conditions in childhood, deficits in social support, or dysfunctional patterns of interpersonal relationships may persist and be reproduced across subsequent stages of life. As a result, even improvements in material conditions in adulthood do not necessarily lead to significant changes in lifestyle or functioning if non-adaptive behavioral and relational patterns remain entrenched. From a life-course perspective, health is therefore not the result of isolated factors acting at a single point in time, but rather the outcome of their accumulation and mutual interaction. This underscores the need to consider the interplay between social, economic, and psychosocial determinants, which, consistent with the previous sections, form a complex system influencing health and wellbeing.

At the same time, it should be noted that many studies focus on determinants of health at a specific point in life, which may limit a comprehensive understanding of aging processes. In contrast, the life-course perspective captures their dynamic and continuous nature, taking into account both early-life experiences and their long-term consequences. Overall, these findings suggest that healthy aging is a complex process embedded in time and social context, in which the accumulation of experiences and the persistence of behavioral patterns play a central role.

### Social interventions and programs

Health is shaped at the individual level, through lifestyle, but macrosocial, macro-economic and structural conditions will also not go unchallenged on the population's health profile. A cross-sectional study conducted in 18 OECD group countries between 2015 and 2019 identified key policies for healthy aging by gender (50). An example of programs that are working well is the National Action Plan for Policies for the Elderly in Sweden. It enables older people to live independently, access quality care and social services, and have good housing, transportation services, and home help. The Nether-lands, meanwhile, has a program involving eight geriatric networks with 650 organizations. High social expenditures of more than 25% of GDP were reported in Denmark, Sweden, and Norway, a rate that exceeds the GDP average for OECD countries. The overall aging index was favorable for men, with a difference of 8.73 points on average (95% CI 7.19–10.3; *p* < 0.0001) compared to women. High scores for all genders were observed in countries with high GDP expenditures on social spending (Denmark, Sweden, Finland, and Norway), while countries such as Hungary, Poland, and Slovenia performed worse (50). The authors of the study suggest that the observed gender differences are due to favoritism toward men in key aspects of aging. Overall, the findings suggest that gender needs should be considered when developing policies and programs for aging populations. Older women suffer from more chronic diseases and disabilities, increasing the need for health care spending.

An interesting initiative that considers the needs of an aging population and ad-dresses the resulting challenges was launched in Barcelona (58). The activity, dubbed “Every Walk You Take,” aimed to collaboratively design and test a prototype of a mobile application that would assist older people in undertaking daily physical activity. The app prototype was developed in collaboration with stakeholders in Barcelona. It recommended routes in the city that older people could take as part of their daily physical activity, considering environmental variables such as air quality, weather, and the difficulty of the route, distance, or geolocation. The application was tested by 21 re-searchers in poor neighborhoods in Barcelona. Barriers and factors that could affect active living were considered. The “Every Walk You Take” initiative is a practical solution using information technology to promote physical activity among older adults and thus help them take care of their health (58).

As an example of an attempt to implement information technology in health management, a focus group study presented the perspectives of various stakeholders on the acceptability and usefulness of technological solutions for bone fragility screening. The opinions of older adults, health and social care professionals, and caregivers in Italy, Poland, and the United Kingdom were collected (59). The goal of the analyses was to understand the facilitators and barriers to technology adoption for frailty screening and management. Stakeholders recognized the potential benefits of using such technology, but its cost was identified as a significant barrier to adoption. On the other hand, technology was perceived to have emotional benefits, such as reducing feelings of isolation.

Evidence from cross-national studies can inform intervention and policy design by highlighting how macro-structural determinants shape quality of life. A longitudinal study of older adults in Portugal, Spain, and Sweden explains how macro-structural determinants can shape quality of life (60). Mean quality of life was highest in Sweden (38.5 ± 5.4) and lowest in Portugal (31.9 ± 5.1). After four years of follow-up, an increase in mean quality of life was observed (32.3 vs. 33.1, *p* < 0.001), while mean quality of life scores decreased in Spain (36.0 vs. 35.2, *p* < 0.001) and remained stable in Sweden (39.2 vs. 39.1, *p* = 0.408). The increase in quality of life in Portugal may be related to the social and economic situation. On the one hand, Portugal was hit by two major adverse events: the global financial crisis of 2008–2009 and the euro crisis of 2010–2012. These crises, characterized by falling GDP, high unemployment, and rising government debt, fostered an unequal distribution of power, status, and resources, which affected people's lives and, consequently, seriously threatened the psychological wellbeing of older population. However, the potential effects of the aforementioned crises could have been minimized through social support from family, friends, or other close people. Similar crises affected Spain, which unfortunately did not deal with their consequences as quickly as Portugal. In addition, Spain fared worse than Portugal in terms of employment for people aged 50 and older, which may also have affected older people's assessment of their quality of life (60).

A study assessing socioeconomic inequalities in chronic diseases and behavioral risk factors among European adults aged 50 and older between 2004 and 2015 found variation in health inequalities by GDP (61). European countries with higher social welfare spending had lower educational inequalities in health. This fact may be directly related to the health profile of the population: more educated people have better information-gathering skills, which increases the likelihood that they will recognize and re-port symptoms of illness and access health services for prompt treatment more quickly.

From a broader perspective, macrosocial and structural conditions emerge as a crucial extension of the individual and psychosocial determinants discussed previously. Factors such as social policies, levels of public expenditure, economic context, and the organization of healthcare systems create the framework within which individuals are able, or limited, to maintain health and activity in older age.

The findings indicate that countries characterized by higher levels of social protection and better access to public services tend to achieve more favorable health and quality-of-life outcomes among older populations. At the same time, significant inequalities persist, both between countries and across social groups, including gender-based disparities, highlighting the need for more targeted and inclusive policy approaches.

Moreover, the growing role of innovative solutions, such as digital technologies and community-based interventions promoting physical activity, suggests new pathways for supporting healthy aging. However, their effectiveness depends on accessibility, acceptance, and alignment with the needs of older individuals. Importantly, these results reinforce the notion that health in older age is not solely determined by individual behaviors or interpersonal relationships, but is deeply embedded in broader structural and economic conditions. These macro-level determinants remain closely interconnected with the previously discussed domains, jointly forming a complex, multilevel system that shapes opportunities for social participation, access to resources, and overall wellbeing in later life.

## Discussion

This systematic review identified several key determinants of healthy aging, including social support, social integration, socioeconomic status, autonomy, and broader structural conditions. Across the included studies, these factors consistently interacted to shape physical, mental, and cognitive health outcomes in older adults, highlighting the multidimensional nature of aging and the importance of both individual and contextual influences.

The findings of this review confirm and extend the theoretical frameworks introduced earlier, particularly the WHO's concept of healthy and active aging and the ecological model of aging (7, 61). The WHO framework emphasizes optimizing opportunities for health participation and security to enhance quality of life, while the ecological model of aging highlights the interaction between individual competence and environmental demands (62). Together, these frameworks underscore the importance of supportive environments that adapt to the changing capacities of older adults and reduce environmental barriers to participation and independence. Beyond these theoretical perspectives, the present synthesis demonstrates that healthy aging is strongly shaped by broader structural and contextual conditions. The reviewed evidence suggests that aging experiences may differ depending on welfare systems, social policies, and characteristics of the local environment. For example, variations between European welfare regimes, such as the universalist Nordic model and the more familistic Southern European model, may influence access to formal care, social resources, and support systems, thereby affecting opportunities for healthy aging. Similarly, neighborhood characteristics, including green spaces, transport accessibility, walkability, and social cohesion, interact with individual capabilities to shape opportunities for social participation and autonomy. Gender-related socioeconomic inequalities also emerged as important contextual factors. These findings support the present review by emphasizing that healthy aging should be understood as a multidimensional process integrating social, psychological, and environmental factors. Women, who are more likely to experience lower pensions, interrupted employment trajectories, and caregiving burdens, may face cumulative vulnerabilities that negatively affect health and quality of life in older age. These findings highlight the importance of linking micro-level determinants, such as social ties, autonomy, and social participation, with macro-level structures, including welfare systems, social policies, and environmental conditions, in order to better understand healthy aging.

Based on the patterns and relationships identified across the included studies, several practical implications can be discerned for initiatives aimed at supporting older individuals and enhancing their health. The findings consistently highlighted the role of psychosocial, socio-economic and structural factors in shaping health and wellbeing in later life. As shown in several studies included in the review, later life often involves transitions such as retirement or widowhood, which may increase vulnerability to social and emotional challenges. A holistic view of health therefore requires coordinated policies that promote cognitive functioning, active lifestyles, and autonomy. Given demographic trends, the existing family-based model of care may become un-sustainable, which makes it necessary to consider greater involvement of formal or supplementary care providers. Because loss of autonomy is often perceived as a marker of advanced aging, initiatives that support independence across various dimensions are essential. Consequently, policy implications should address the structural determinants of health by fostering age-friendly environments, mitigating socioeconomic disparities, and endorsing gender-sensitive strategies to promote healthy aging. These initiatives necessitate cross-sectoral collaboration to ensure equitable access to resources and opportunities for active aging.

Emphasizing neighborhoods and building inclusive communities is also key. Professionals and policymakers should consider psychosocial aspects of wellbeing - such as satisfaction, autonomy, and meaningful engagement – alongside physical health and functional ability. Evidence from studies conducted among older adults in China further highlights the importance of psychosocial dimensions of healthy aging. Community engagement, social support, mindfulness, and positive emotional experiences were shown to be associated with lower levels of loneliness and higher life satisfaction among retirees. Although these findings originate from a different cultural context, they suggest that emotional and social wellbeing may represent universal components of healthy aging across diverse populations (63–65). Active aging strategies that improve quality of life include opportunities for participation in community life, continued learning, physical activity, and support for managing daily tasks. Incentives to remain economically active are also beneficial, as work helps maintain internal and external resources and enhances a sense of purpose. Flexible working conditions further support vitality by enabling a balanced allocation of time between work and leisure.

Evidence from the reviewed studies suggests that interventions such as community-based support groups, age-friendly urban planning, and digital literacy training for older adults can significantly enhance social participation and autonomy. Furthermore, the reviewed studies indicate that policies promoting flexible retirement options and lifelong learning opportunities may facilitate the continued economic and social engagement of older adults. Future research should prioritize longitudinal studies capable of capturing the dynamic and cumulative nature of aging processes and social determinants of health over time. Greater attention should also be paid to intersectional inequalities, particularly those related to gender, socioeconomic status, and place of residence. Comparative analyses of rural and urban contexts, as well as differences between European welfare systems, may provide a more nuanced understanding of aging experiences and structural inequalities. Such research would strengthen the evidence base for developing integrated public health policies addressing both the individual and structural dimensions of healthy aging.

Additionally, future evidence syntheses could be complemented by bibliometric approaches using software such as CiteSpace, VOSviewer, or similar tools. Such methods may facilitate visualization of research trends, thematic clusters, and collaboration networks within the field of healthy aging. However, the absence of bibliometric analysis does not constitute a methodological limitation of the present review, as the primary objective was a qualitative synthesis of evidence rather than mapping scientific productivity or citation patterns.

## Limitations

Some methodological limitations should be noted in the study presented here. Due to the selection of only three electronic databases, the narrowed time frame (from 2019 to 2024), and full English-language texts, other important publications that could alter the analysis of the results may have been omitted. In addition, the time range included in the study (2019–2024) included the COVID-19 pandemic state (2019–2023), during which social isolation was implemented in most European countries. The specifics of this period may be a limitation. However, given the social and systemic changes that followed, it might be appropriate to look at the findings presented in a long-term con-text. Furthermore, variability in definitions of healthy aging and active aging, as well as heterogeneity in measurement tools across studies, may affect comparability and precision of results. The use of the “free full text” filter in literature databases may have introduced selection bias; however, it ensured full access to study details necessary for accurate narrative synthesis and evaluation of methodological quality. A notable limitation is the predominance of cross-sectional and qualitative studies, which constrains the ability to infer causality. Additionally, a substantial proportion of studies were evaluated as having a moderate or high risk of bias, particularly due to selection issues and a lack of transparency in data collection. We did not perform sensitivity analyses or re-synthesize results after excluding studies with a high risk of bias, as the review was qualitative and exploratory in nature, aiming to capture a broad range of perspectives rather than estimate quantitative effects. This may impact the robustness of the conclusions. Many of the included studies did not provide country-specific data, making it impossible to consistently distinguish regional differences; therefore, the term “Europe” was applied as a collective reference. Furthermore, the review focused exclusively on studies conducted in European populations. Consequently, potentially valuable evidence from other cultural and social contexts was not included. Research conducted among older adults in Asian countries, including China, suggests that factors such as mindfulness, social support, community engagement, and emotional well-being may influence healthy aging in ways that could complement the European perspective (63, 64). Therefore, caution should be exercised when generalizing the present findings beyond the European context. This limitation should be considered when interpreting the findings, as structural and cultural variations across European regions may influence aging experiences.

## Conclusions

The findings of this review indicate that social capital, social support, social integration, and the absence of loneliness play crucial roles in maintaining health and cognitive functioning among older adults. Engagement in social, physical, and occupational activities appears to support the preservation of internal and external resources, enhance the sense of purpose in life, and contribute to better overall health outcomes. Maintaining independence and autonomy is essential for the wellbeing of older adults, as these factors strengthen their sense of control and positively influence their quality of life. These findings suggest that policies aimed at promoting healthy aging should adopt a holistic approach that integrates the physical, psychosocial, and social dimensions of health to effectively support the wellbeing of older adults.

## Data Availability

The datasets presented in this article are not readily available because no new datasets were generated or analyzed in this study.
