# Supplementary material for: Psychosocial and economic factors related to better health among older adults in Europe – a systematic review
Source: Front Public Health. 2026 Aug 6;14:1858548. doi: 10.3389/fpubh.2026.1858548 (PMC13489839; doi:10.3389/fpubh.2026.1858548)
Supplement: Supplementary file 2 [file Supplementary_file_2.docx]

Supplement B. Risk of bias assessment

| No | First Author | Study Type | Risk of Bias Tool | Risk of Bias Assessment | Comments |
| --- | --- | --- | --- | --- | --- |
| 1 | Silberman-Beltramella | cross-sectional | NOS | Moderate | Some concerns about selection bias. |
| 2 | Carstensen | qualitative | CASP | High | Limited transparency in data collection. |
| 3 | Fastame | cross-sectional | NOS | Moderate | Some concerns about selection bias. |
| 4 | Christiansen | cohort study | NOS | Low | Well-conducted study with minimal bias. |
| 5 | Brown | qualitative research | CASP | High | Limited transparency in data collection. |
| 6 | Karma | qualitative research | CASP | High | Limited transparency in data collection. |
| 7 | Sialino | qualitative research | CASP | High | Limited transparency in data collection. |
| 8 | Bertogg | cohort study | NOS | Low | Well-conducted study with minimal bias. |
| 9 | Hackert | qualitative research | CASP | High | Limited transparency in data collection. |
| 10 | Aliberti | cross-sectional | NOS | Moderate | Some concerns about selection bias. |
| 11 | Gao | cohort study | NOS | Low | Well-conducted study with minimal bias. |
| 12 | Luo | cross-sectional | NOS | Moderate | Some concerns about selection bias. |
| 13 | Siqeca | cross-sectional | NOS | Moderate | Some concerns about selection bias. |
| 14 | Guastafierro | cross-sectional | NOS | Moderate | Some concerns about selection bias. |
| 15 | León | Structural equation modelling | Other | Moderate | General concerns about study design. |
| 16 | Bosch-Farré | qualitative research | CASP | High | Limited transparency in data collection. |
| 17 | Mikelsone | questionnaire survey | CASP | High | Limited transparency in data collection. |
| 18 | Henriques | cross-sectional | NOS | Moderate | Some concerns about selection bias. |
| 19 | Ophey | cross-sectional | NOS | Moderate | Some concerns about selection bias. |
| 20 | Kovacs | cross-sectional | NOS | Moderate | Some concerns about selection bias. |
| 21 | Calderón-Larrañaga | questionnaire | CASP | High | Limited transparency in data collection. |
| 22 | Alonso-Perez | cohort study | NOS | Low | Well-conducted study with minimal bias. |
| 23 | Nivestam | cross-sectional | NOS | Moderate | Some concerns about selection bias. |
| 24 | Maharani | cohort study | NOS | Low | Well-conducted study with minimal bias. |
| 25 | Chen | cross-sectional | NOS | Moderate | Some concerns about selection bias. |
| 26 | Mikkola | cohort study | NOS | Low | Well-conducted study with minimal bias. |
| 27 | Bjuhr | qualitative | CASP | High | Limited transparency in data collection. |
| 28 | Wang Y | cohort study | NOS | Low | Well-conducted study with minimal bias. |
| 29 | Hartig | cross-sectional | NOS | Moderate | Some concerns about selection bias. |
| 30 | Cassarino | questionnaire | CASP | High | Limited transparency in data collection. |
| 31 | Zhang | cohort study | NOS | Low | Well-conducted study with minimal bias. |
| 32 | Yang | retrospective cohort analysis | NOS | Low | Well-conducted study with minimal bias. |
| 33 | Naik | Intervention study | Other | Moderate | General concerns about study design. |
| 34 | Gwyther | qualitative | CASP | High | Limited transparency in data collection. |
| 35 | Ayala | cohort study | NOS | Low | Well-conducted study with minimal bias. |
| 36 | Bono | cohort study | NOS | Low | Well-conducted study with minimal bias. |
