# Supplementary material for: Psychosocial and economic factors related to better health among older adults in Europe – a systematic review
Source: Front Public Health. 2026 Aug 6;14:1858548. doi: 10.3389/fpubh.2026.1858548 (PMC13489839; doi:10.3389/fpubh.2026.1858548)
Supplement: Supplementary file 3 [file Supplementary_file_3.docx]

| 1 | Silberman-Beltramella | cross-sectional | 5583 people | 55+ | Spain | social relationships | The number of friends, family members, and satisfaction with social network were key factors affecting physical, emotional, functional, and cognitive/sensory health. | 6 |
| --- | --- | --- | --- | --- | --- | --- | --- | --- |
| 2 | Carstensen | qualitative | 17 men | 85-90 | Sweden | financial independence, social contacts, friendships | Independence and close relationships, and a sense of belonging were key aspects of healthy ageing | 8 |
| 3 | Fastame | cross-sectional | 180 people | 65-94 | Italy | family ties | Satisfaction with family relationships was an important factor in healthy ageing | 9 |
| 4 | Christiansen | cohort study | 24,687 people | 35-79 | Denmark | loneliness, social isolation | Loneliness was independently associated with a higher risk of cardiovascular disease and type diabetes | 18 |
| 5 | Brown | qualitative research, in-depth interviews | 33 people | 70+ | UK | loneliness, isolation, social ties | Support from neighbours and local communities was key to maintaining the mental and physical health of older adults during the lockdown | 21 |
| 6 | Karma | qualitative research, in-depth interviews | 12 people | 70+ | Switzerland | social belonging, social inclusion | Emphasising community-based approaches can create a supportive environment for older adults. Home visits by specialized nurses focused on health risks and disabilities can be an appropriate approach to support the older population effectively. | 24 |
| 7 | Sialino | qualitative research, semi-structured interviews | 34 women | 55+ | Netherlands | social support, social relationships, social pressure | Motivators and barriers to healthy lifestyles, as well as perspectives on healthy ageing, vary by lifestyle. Personal health is a common motivator in all lifestyles. An important motivator in active lifestyles was peer pressure and support. | 25 |
| 8 | Bertogg | cohort study | 81,814 people | 50+ | 20 European countries (SHARE survey) | education, partnership (no loneliness) | Living in a partnership was beneficial for cognitive health in later life. Socioeconomic status plays a key role in cognitive ageing. Individuals with higher cognitive reservoirs and better socioeconomic conditions experienced less cognitive decline. | 26 |
| 9 | Hackert | qualitative research | 53 people | 65+ | Netherlands | family, support, social relationships, independence | Among the most important factors affecting the well-being of older adults are health, social relationships, independence, and a sense of security | 27 |
| 10 | Aliberti | cross-sectional | 1200 | 65-89 and 90-115 | Italy | social support, social integration | Individuals who had strong social support networks and were more socially engaged showed better health outcomes | 28 |
| 11 | Gao | cohort study | 15,066 participants in the U.S. and 10,290 in the UK. | mean age = 66.1 years, SD = 9.8 in the U.S. and 66.9 years, SD = 10.5 in UK.. | UK, USA | loneliness, social involvement | Older adults who experienced greater social isolation had higher levels of inflammatory markers. Regular participation in social activities was associated with lower levels of inflammation | 29 |
| 12 | Luo | cross-sectional | 103 people | 65-84 | Switzerland | social contacts, social involvement | Regular participation in social activities helped maintain higher levels of emotional well-being | 33 |
| 13 | Siqeca | cross-sectional | 8786 people | 75+ | Switzerland | social involvement, education, socioeconomic status | Active participation in social activities was associated with improved HRQoL, higher SES was associated with better health-related quality of life. | 34 |
| 14 | Guastafierro | cross-sectional | 431 people | 50+ | Italy | social support, quality of life | Social support was a key factor in the health and well-being of the elderly. | 35 |
| 15 | León | Structural equation modelling | 128 people | 60+ | Spain | social support, social capital | The availability of social support improves quality of life and explicit memory, and reduces depression in active elderly people. The availability of social resources, defined as people with whom one interacts daily, other family members, close friends, or institutions that can help in an emergency, allows people to avoid isolation and loneliness, increasing satisfaction and well-being | 36 |
| 16 | Bosch-Farré | qualitative research, focus groups | 71 people | 62-92 | Spain | family environment, financial resources | Physical and mental health, family environment, and financial stability were key elements of quality of life. Perceptions of the role of the elderly in the community depended on their age, health, and attitude toward life | 37 |
| 17 | Mikelsone | questionnaire survey - direct interview CAPI | 50 people | 50+ | Lithuania, Latvia, Estonia | social activity, feelings of loneliness, isolation, education, unemployment | Economic factors such as income, retirement, and financial security play a significant role in healthy ageing. The environment, including quality of housing, neighbourhood safety, and access to social services, has a significant impact on healthy ageing. | 38 |
| 18 | Henriques | cross-sectional | 585 people | 60-84 | Portugal | social support | Those with higher levels of social support had better mental health and overall well-being scores. Older adults who had active social lives reported better mental health and fewer feelings of isolation. | 39 |
| 19 | Ophey | cross-sectional | 1503 people | 80+ | Germany | education, social engagement | Younger age, more years of education, and more cognitive lifestyle activities were identified as the most consistent determinants of both better cognitive and behavioural outcomes | 40 |
| 20 | Kovacs | cross-sectional | 860 people | 18+, with a separate group of 75+ | Hungary | social support, financial resources | self-assessment of financial status, social support, sense of control over one's health, activity limitation, and pain intensity | 41 |
| 21 | Calderón-Larrañaga | questionnaire | 3108 people | 60+ | Sweden | social support, education, and financial resources | Insufficient physical activity, financial burden, education <12 years, low social connectedness, and low social participation were associated with belonging to average/worst trajectories. Health | 42 |
| 22 | Alonso-Perez | cohort study | 19953 people | 50-85 | data from 13 European countries (SHARE survey) | social activity, family | Transitioning to the role of grandparents was associated with higher levels of cognitive functioning, especially in women. Social support played a key role in improving cognitive health. Older adults who actively participated in their grandchildren's lives reported better mental health and fewer feelings of isolation. | 43 |
| 23 | Nivestam | cross-sectional | 619 people; | Mean age: 80.6 | Sweden | social involvement | The ability to do activities that make a person feel valuable and the absence of physical problems that affect participation in social activities were important factors in good health. | 44 |
| 24 | Maharani | cohort study | 6735 people | 50+ | UK and Indonesia | income, education, and social support | In the UK, people with secondary or higher education had lower CRP levels. Social support played a key role in the health of the elderly, affecting CRP levels and overall health. In the UK, people with higher levels of wealth had lower CRP levels. | 45 |
| 25 | Chen | cross-sectional | Analysis of population data from 18 OECD countries, a lack of accurate data on the number of subjects studied | 65+ | 18 OECD countries | structural determinants, engagement | Men showed higher scores in productivity, engagement, social cohesion, and economic security domains than women | 46 |
| 26 | Mikkola | cohort study | 13140 people | Born in 1934-44 | Finland | social support, mental activity, income, education | The probability of healthy survival, as the upper limit of healthy ageing, was less than 50% from age 65. The probability of healthy survival and healthy ageing depended on several factors throughout the life cycle. | 47 |
| 27 | Bjuhr | qualitative, semi-structured interviews | 18 people | 67-90 | Sweden | working life, social activity | Working after 65 years of age brought economic benefits, contributed to the maintenance of external resources such as social enrichment and better daily routines, added meaning to life, making the elderly feel needed. | 48 |
| 28 | Wang Y | cohort study | 69 160 people | 50+ | 24 countries, including non-European: analysis of survey data Health and Retirement Study (HRS); English Longitudinal Study of Ageing (ELSA); Survey of Health, Ageing and Retirement in Europe (SHARE); China Health and Retirement Longitudinal Study (CHARLS); and Mexican Health and Ageing Study (MHAS). | SES social activity and loneliness | Individuals with higher levels of education and better financial resources had a lower risk of depression. Higher levels of loneliness were strongly associated with higher levels of depressive symptoms, while regular participation in social activities was associated with lower levels of depressive symptoms. | 49 |
| 29 | Hartig | cross-sectional | 2899 people | 45-75 | Germany | neighbourhood environment, social integration, SES, education | The socioeconomic status of the neighbourhood affects the occurrence and intensity of positive affect. Higher levels of social integration were associated with higher positive affect scores | 50 |
| 30 | Cassarino | questionnaire | 112 people | 60+ | Ireland | neighbourhood environment, infrastructure, availability of green space | A complex relationship was observed between individual cognitive/sensory needs (stimulation-seeking vs. avoidance), preferences for social interaction, and place of residence in relation to the practice of walking. Walking preferences differed according to the purpose of the walk (recreation vs. transportation). | 51 |
| 31 | Zhang | cohort study | 160,000 people | 50-80 | UK | social (emotional) support, life cycle perspective | High levels of emotional support and strong social relationships in childhood were associated with better mental and physical health in adulthood. | 52 |
| 32 | Yang | retrospective cohort analysis | 127,495 people | 40-69 | UK | childhood difficulties, including physical neglect, emotional neglect, sexual abuse, physical violence, and emotional abuse, or lifestyle, life cycle perspective | Individuals who experienced childhood difficulties (violence, neglect, lack of support) had a higher risk of accelerated ageing. | 53 |
| 33 | Naik | Intervention study using mobile technology (mHealth) | 124 people | 55+ | Spain | Barriers and facilitators to active living, social determinants of health, and community health resources | The study highlights the importance of mobile technology and community initiatives in promoting healthy ageing through increased physical activity and community engagement | 54 |
| 34 | Gwyther | qualitative, focus groups | 99 healthy and frail/pre-frail older adults, health and social care providers, and caregivers | 65+ | Italy, Poland, UK | social support, experience with technology | Technology for frailty management was considered valuable by professionals and caregivers, but older adults had concerns about its availability and usefulness. Social support and previous experience with technology were key factors in older adults' acceptance of technology | 55 |
| 35 | Ayala | cohort study | 7589 people | 50+ | Portugal | structural determinants (political, economic), social participation | A significant interaction was found between the country-social participation component. In Spain, higher levels of participation were associated with greater improvements in quality of life after 4 years, but not in Sweden or Portugal. The components of physical and emotional health, functional fitness, and cognitive and sensory fitness were associated with changes in quality of life over time in all countries. | 56 |
| 36 | Bono | cohort study | 25,016 people in 2004 and 43,916 in 2015 | 50+ | Austria, Germany, France, Switzerland, Belgium, Denmark, Sweden, Spain, Italy, Greece | SES, marital status | Among the determinants of health, socioeconomic status (SES), education, and marital status played the greatest role. | 57 |
